# Polymer/Metal Organic Framework (MOF) Nanocomposites for Biomedical Applications

**DOI:** 10.3390/molecules25010185

**Published:** 2020-01-01

**Authors:** Dimitrios Giliopoulos, Alexandra Zamboulis, Dimitrios Giannakoudakis, Dimitrios Bikiaris, Konstantinos Triantafyllidis

**Affiliations:** 1 Laboratory of Chemical and Environmental Technology, Department of Chemistry, Aristotle University of Thessaloniki, GR-54124 Thessaloniki, Greece; dagchem@gmail.com; 2 Laboratory of Polymer Chemistry and Technology, Department of Chemistry, Aristotle University of Thessaloniki, GR-54124 Thessaloniki, Greece; azampouli@chem.auth.gr

**Keywords:** metal organic framework, polymer nanocomposites, drug delivery, magnetic resonance imaging

## Abstract

The utilization of polymer/metal organic framework (MOF) nanocomposites in various biomedical applications has been widely studied due to their unique properties that arise from MOFs or hybrid composite systems. This review focuses on the types of polymer/MOF nanocomposites used in drug delivery and imaging applications. Initially, a comprehensive introduction to the synthesis and structure of MOFs and bio-MOFs is presented. Subsequently, the properties and the performance of polymer/MOF nanocomposites used in these applications are examined, in relation to the approach applied for their synthesis: (i) non-covalent attachment, (ii) covalent attachment, (iii) polymer coordination to metal ions, (iv) MOF encapsulation in polymers, and (v) other strategies. A critical comparison and discussion of the effectiveness of polymer/MOF nanocomposites regarding their synthesis methods and their structural characteristics is presented.

## 1. Introduction

The homogeneous dispersion of inorganic, organic, or hybrid nanoscale components inside a polymeric matrix results in materials with physically and/or chemically distinct phases that are called polymer nanocomposites. Polymer nanocomposites have unique or improved properties when compared to pristine polymers or conventional composites and these properties can easily be tuned by controlling the type or the concentration of the additives, selecting specific production methods, and functionalizing the surface of the additives, etc. [1,2,3,4,5,6,7,8]. Due to the superior properties and the diversity of products, polymer nanocomposites are used in a variety of applications in most industrial and research fields. Among them, biomedicine has greatly benefited from the progress in nanocomposite materials regarding the advances that have been made in the areas of diagnosis, monitoring, and therapy. Some of the biomedical applications of polymer nanocomposites may include drug or gene delivery, skin regeneration, soft-tissue engineering, bone or joint replacement, bioimaging, biosensors, dental or antimicrobial applications, and many other [9,10,11].

Many types of nanostructured materials have been used in combination with biocompatible polymers to produce nanocomposites for biomedical applications such as clays, carbon nanotubes, graphene, metal oxides, porous nanomaterials, magnetic nanoparticles, and others. As part of more complex systems for biomedical applications, nanostructured materials may exhibit various functions. For example, they can reinforce the polymer matrix or offer some new property, they can interact with a substrate or a substance when it would be impossible for the polymer, and they can control the transport phenomena through the polymer matrix, etc. [10,12,13,14,15,16].

MOFs are a class of crystalline materials possessing structures formed from the coordination of metal ions to multidentate organic groups. The main characteristics of MOFs are the high degree of porosity and the tunable architecture of the structure by selecting appropriate metal ions and linkers. Furthermore, MOFs can have their surface further modified, thereby increasing their functionality. These characteristics make MOFs ideal candidates for biomedical applications like drug delivery and magnetic resonance imaging (MRI) [17,18,19]. As it concerns drug delivery, the high surface areas and large pore sizes of MOFs are favorable for the encapsulation of high drug loadings [20], while the high structural and functional flexibility of MOFs allow their adaption to the shape, size, and functionality of the drug molecules [20,21]. On the other hand, regarding imaging applications, MOFs can be modified with chemical groups and uniquely affect the delivery of contrast imaging agents [22]. Moreover, MOFs have the advantage of acting simultaneously as MRI contrast agents and drug carriers, serving both purposes of diagnosis and therapy [23]. As can be understood, the use of MOFs in biomedical applications offers serious advantages to scientists in the fields of diagnosis, monitoring, and therapy. As a result, numerous studies over the last years have focused on the combined use of MOFs and biocompatible polymers, aiming at the development of more sophisticated systems that would be more effective than previous products while ensuring a higher quality of life for patients.

In this review, we examine the various types of polymer/MOF nanocomposites used in biomedical applications, and more specifically in drug delivery and imaging. Although there have been many reviews covering various aspects of the use of MOFs in biomedical applications, no work at the present has reviewed the composite materials of polymer matrix and drug loaded MOF additives in biomedical applications. More specifically, we focused on the different approaches followed to produce the composites and discuss the findings regarding the behavior of the composites in each application.

## 2. Metal Organic Frameworks

**Figure 1:**
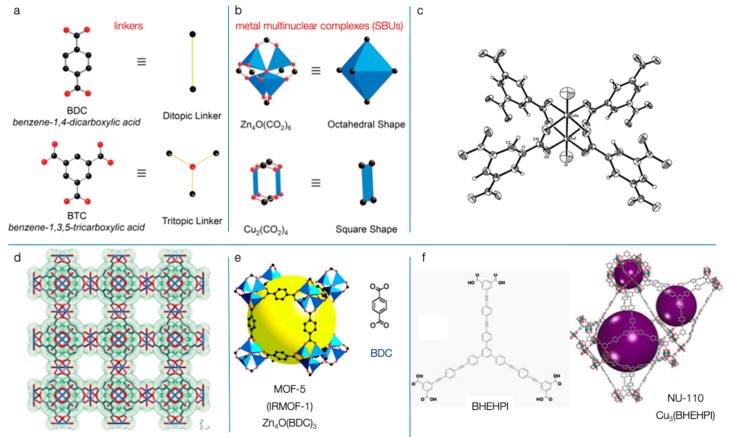
(**a**) The linkers [32]; (**b**) the metal clusters/multinuclear complexes (Secondary Building Units, SBUs) of HKUST-1 and MOF-5 (color assignation: black for C, red for O, blue for Cu squares and Zn polyhedrals; H atoms are omitted) [32]; (**c**) the dicopper(II) tetracarboxylate building block of HKUST-1 [30]; (**d**) the polymeric framework of HKUST-1 (viewed down the direction) [30]; (**e**) the single crystal structure of MOF-5 (the yellow spheres represent the maximum volume of the biggest cavity) [33]; (**f**) the chemical structure of the ligand and the different cages of the NU-110 framework [34].

Metal organic frameworks, also known as porous coordination networks (PCNs) or porous coordination polymers (PCPs), are in general highly porous 1-, 2-, or 3-dimensional extended organic-inorganic coordination structures [24,25]. Their network is composed of metal centers (ions, clusters of ions, or better multinuclear complexes) linked by di- or polydentate organic bridges called linkers (Figure 1a,b). Even though coordination chemistry between metal ions and organic linkers to form coordination polymers (like Werner complexes or Prussian blue compounds) has a prolonged history [26], Hoskins and Robson were the first to suggest in 1989 of the potential synthesis of solid porous polymeric materials based on coordination bonds [27]. The introduction in the literature of the terminology ‘metal organic framework’ occurred in 1995 from Yaghi and Li, who reported the hydrothermal synthesis of a “zeolite-like” crystalline structure by the polymeric coordination of copper with 4,4′-bipyridine and nitrate ions [28]. It took some years in order for this class of new supramolecular materials to become a mainstream topic of research, with the most influential reports published in 1999 for two 3-D frameworks that still act as benchmark representatives [29]: HKUST-1 by Chui et al. [30] and MOF-5 by Li et al. [31] (Figure 1c,d). The former one, known also as MOF-199, took its name from the place of synthesis (Hong Kong University of Science and Technology) and is built up from a paddlewheel shaped Cu_2_(CO_2_)_4_ metal cluster/subunit (called the secondary building unit, SBU) consisting of a dimer of Cu^2+^ ions, where each copper ion has been coordinated with four benzene-1,3,5-tricarboxylic acid (BTC) as a tritopic linker. 3-D illustrations of the structure of the polymeric framework, as reported in the original article, can be seen in Figure 1c,d. MOF-5 (known also as IRMOF-1) has an octahedral multinuclear complex/SBU, Zn_4_O(CO_2_)_6_, in which an O^2−^ ion is tetrahedrally linked with four Zn^2+^ ions, and each zinc ion is coordinated with three oxygens from three different 1,4-benzenedicarboxylate (terephthalate, BDC) linkers, resulting in a cubic framework (Figure 1e).

As S. Kaskel mentions in his book [35], all of the known MOFs up until 2002 could be summarized within a book chapter. Nowadays, there are more than ten thousand 3-D registered MOFs in the Cambridge Structural Database and more than twenty thousand hypothetical or real known MOFs [36]. The research effort continues to be extensive, with many new MOFs commercially available. Due to the high surface area, porosity, and tailorable size of the pores/cages as a result of the diversity of combination of metal and linkers, MOFs have garnered an enormous boost in attention in the last decades for a wide range of potential applications such as adsorption, gas storage, purification, separation, chemical sensing, and even for selective catalytic processes against toxic compounds [24,25,37,38,39,40,41]. The pores/cavities are created as free spaces, cages, or voids inside the structure. The reported surface area values in the initial article for HKUST-1, calculated based on N_2_ adsorption/desorption tests, were 692.2 m^2^ g^−1^ using the Brunauer–Emmett–Teller (BET) equation and 917.6 m^2^ g^−1^ based on the Langmuir approach, while the single-point total pore volume was 0.333 cm^3^ g^–1^ [30]. In the case of MOF-5, the authors reported (based on liquid nitrogen vapor sorption test) an estimated Langmuir surface area of 2900 m^2^ g^−1^ (2320 m^2^ g^−1^ based on the BET method) and a pore volume (based Dubinin–Raduskhvich equation) up to 1.04 cm^3^ g^−1^ [31].

Great effort has been given to achieve higher porosity, predominately by increasing the size of the linkers, leading to significantly higher structural feature values when compared to commonly used activated carbons and zeolites [42,43,44,45,46]. A characteristic example is Cu_3_(BHEHPI) or NU-110 (NU stands for Northwestern University in Chicago, USA), which has the highest reported surface area and total pore volume up to now [34,44]. This copper based MOF (Figure 1f) was reported by O. Farha, J. Hupp, and co-workers in 2012 [31], where a hexacarboxylate macromolecule was used as a ligand (BHEHPI– stands for 5,5′,5″-((((benzene-1,3,5-triyltris(benzene-4,1-diyl)) tris(ethyne-2,1-diyl))-tris(benzene-4,1-diyl)) tris(ethyne-2,1-diyl)) triisophthalate). The reported BET surface area by N_2_ sorption experiments was 7140 m^2^ g^−1^ and the total pore volume was 4.4 cm^3^ g^−1^, values that are the highest experimentally obtained up today. Interestingly, the obtained nitrogen isotherm was closer to type-IV rather than to type-I and revealed multiple sizes of pores, a fact that is consistent with the different types of illustrated cages in Figure 1f. The authors also showed that in general, the theoretical surface of the MOFs could reach up to 14,600 m^2^ g^−1^ [34].

An important factor that should be taken into consideration in the design and synthesis of MOFs for application in aquatic environments is that their stability depends on the strength of coordination between the metal and linker [47]. The reason behind this instability is the ability of water to interact with the metal ions/clusters competitively to the linkers, leading to the collapse of the framework. There are also various other factors that play a crucial role in the stability of the MOFs, with the most important being crystallinity, hydrophobicity, and the extent of the defectous sites [43]. Additionally, the temperature and pH should also be considered. In general, the hard/soft acid/base (HSAB) principles can predict the level of metal/linker coordination strength [48,49]. Hard acidic metal ions (like Zr^4+^, Cr^3+^, Al^3+^, and Fe^3+^) combined with carboxylate-based linkers acting as hard bases result in frameworks with a significant water resistivity/stability. Stability against water is also due to the coordination between weak acidic metal ions (like Cu^2+^, Mn^2^, Zn^2+^, Ag^+^, and Ni^2+^) and linkers with a weak basic character (like pyrazolates, triazolates, and imidazolates). Combining strong acidic metal ions with weak basic linkers, and vice versa, results in a vulnerability to water frameworks.

### 2.1. Biological Metal Organic Frameworks (BioMOFs)

In the last decade, a novel and attractive sub-class of MOFs, the biological metal organic framework (BioMOFs), has had an augmented degree of interest, giving rise to new opportunities for their utilization in a plethora of biological and medical applications. Although there is no specific definition for these new generation biocompatible materials, in order for an inorganic–organic framework to be classified as a BioMOF, it should either consist of at least a biomolecule or have a direct application across medicine and biology. With the exception of biocompatibility, the other two are features of utmost importance with regard to BioMOFs design are to possess the appropriate size of pores/cages and to be able to selectively and strongly retain the targeted therapeutic/drug. The latter aspect is known as host–guest chemistry, which is critical for supramolecular recognition features. The most important fields of BioMOF utilization can be summarized as adsorption/encapsulation, the protection and delivery of molecular therapeutics (drug delivery), enantioseparation, magnetic resonance imaging (MRI), photothermal therapy, biomimetic catalysis, biobanking, biosensing, and cell and various manipulations, etc. [50,51].

**Figure 2:**
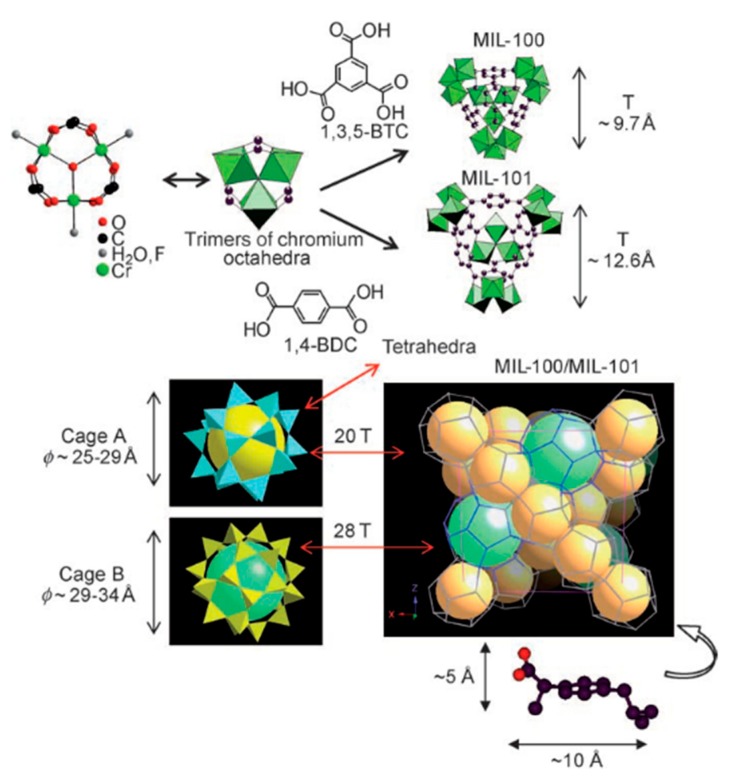
Schematic 3-D representation of the tetrahedra (T) consisting of trimers of chromium octahedra and 1,3,5-benzene tricarboxylic acid (BTC) or 1,4-benzene dicarboxylic acid (BDC) in MIL-101 and MIL-100, respectively (**top**) and a schematic 3-D illustration of the zeotype-architecture MIL-100 and MIL-101 (**bottom**) [53].

Prior to the appearance of the BioMOF, drug delivery methods were based on two routes. In the first and “organic route”, a biocompatible host (such as polymers or dendritic macromolecules) was used as the host. Even though it is possible to encapsulate a wide range of therapeutics via the organic route, the controlled release is challenging due to there being no well-defined porosity or a homogeneous distribution of the drug inside the host matrix [52,53,54]. For the second and “inorganic route”, a mesoporous inorganic substance (like silicate or zeolite) acts as the host through grafting of the pore’s walls, leading to a lowering of the porosity and the therapeutic-loading capacity [55,56]. In 2006, the innovative work of Horcajada et al. [53] introduced a “hybrid route”, in which a MOF structure was utilized as the host. They synthesized two cubic zeotypic MOFs, abbreviated as MIL-100 and MIL-101 (MIL, Materials Institute Lavoisier). MIL-100 and MIL-101 were built from trimers of chromium octahedras and di-(1,3,5-benzene tricarboxylic acid, BTC) or tri-carboxylic acid (1,4-benzenedicarboxylic acid, BDC), respectively (Figure 2). MIL-100 showed pore/cage sizes between 25–29 Å and a specific surface area of 3340 m^2^ g^−1^, while the respective values for MIL-101 were reported as 29–34 Å and 5510 m^2^ g^−1^. The material showed a remarkably great capacity toward ibuprofen, reaching a loading of 1.4 g per one gram in the case of MIL-101 [53]. Even though this study was criticized due to the known toxicity of Cr, it opened the road for many other MOFs to be designed and tested as hosts for controllable drug delivery. Interestingly, in 2010, the same team showed that analogue structured MOFs could be obtained based on Fe in aqueous or ethanolic solutions, even by avoiding the use of other organic solvents and chromium [21]. The low toxicity of these BioMOFs was demonstrated by in vivo rat and in vitro cell studies. The nanoscaled Fe-MIL-100 showed a 31.9% loading per weight for the antitumoral drug, busulfan, a value five-fold higher than that of the existing busulfan delivery platforms and with a similar cytotoxic activity as the free drug. Additionally, loading with the anti-HIV agent (AZT-TP) was revealed as promising for the “in vitro inhibition of virus replication”.

### 2.2. Metal Organic Frameworks (MOFs) for Biomedical Applications

Initially, many of the already known MOFs were examined for potential bio-applications. However, the modern strategy toward the exploration of novel BioMOFs is the usage of biological molecules as ligands. Even though some biomolecules have been successfully utilized as organic linkers, their complicated chemistry (like molecular symmetry, geometry, flexibility etc.) has hindered the possibilities of obtaining crystalline frameworks with the desired properties. Among the most intensively studied biomolecules are nucleobases, amino acids, peptides and proteins, porphyrins, metalloporphyrins, and cyclodextrin [21,50,57,58]. More details can be found in the very recent comprehensive review by Cai et al. [50].

**Figure 3:**
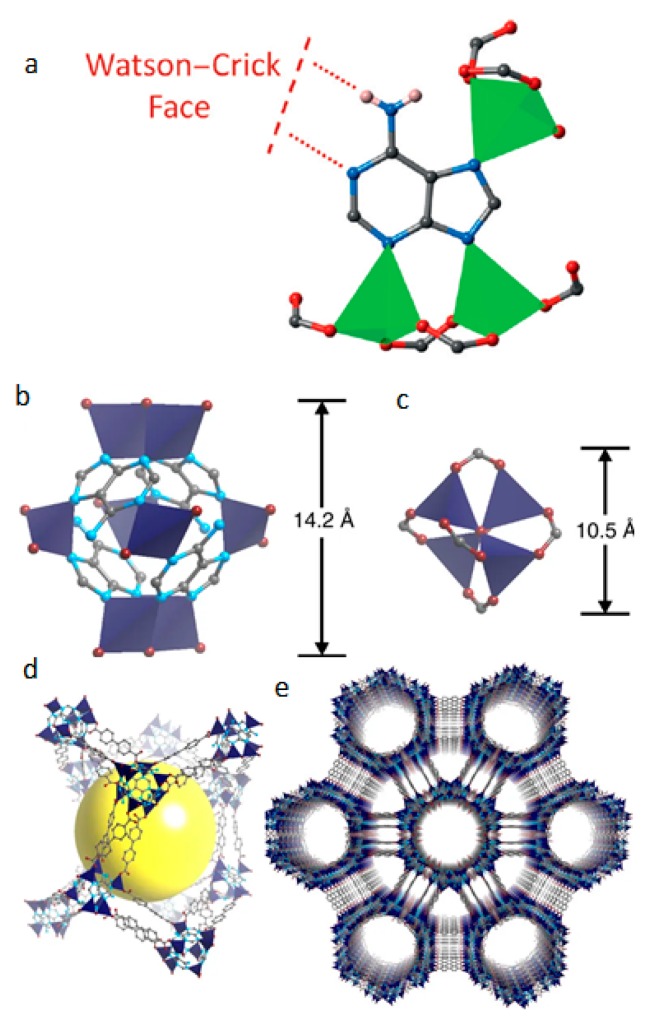
(**a**) Open Watson–Crick sites and the coordination environment of adenine in ZnBTCA [59]. (**b**,**c**) A comparative illustration of the structure and size of the building units in bio-MOF-100 and the basic zinc-carboxylate building [57]. (**d**,**e**) The 3-D crystal structure of bio-MOF-100 where the cavities (yellow sphere) and the large channels can be seen (Zn^2+^: green or dark blue tetrahedra, C: grey spheres, O: red spheres, N: blue spheres, H: omitted for clarity) [57].

Extensive efforts have been given to alternative approaches for the utilization of biomolecules in the MOF matrix. The main concept is to use the biomolecules in addition to conventional linkers, or use combinations of biomolecules and common linkers. An example is the utilization of a symmetric auxiliary molecule in order to compensate the limited symmetry of the biomolecule. ZnBTCA (where BTC stands for benzene-1,3,5-tricarboxyl and A for adenine) is a characteristic paradigm of the utilization of nucleobase moieties, as reported by Cai and co-workers in 2015 [59]. Adeninate moieties were periodically introduced into the framework, providing sufficient and available Watsin–Click faces (Figure 3a). The kinetic and thermodynamic studies revealed unusual hysteresis of the interaction of the Watsin–Click faces with the amino groups of the guest. It was also reported that the combination of adenine and thymine conferred a pronounced adaptive recognition/response.

Another alternative approach is based on the use of asymmetric biomolecules for the formation of a metal–biomolecule cluster as a secondary building unit. In 2012, An et al. reported that zinc-adeninate SBU can be interconnected with a relatively short dicarboxylate linker (biphenyldicarboxylate, BPDC), forming an exclusively mesoporous bioMOF, bio-MOF-100 [57]. This material showed a pioneering high surface area (4300 m^2^ g^−1^) and total pore volume (4.3 cm^3^ g^−1^) as well as very low crystal density (~0.3 g cm^−3^). The structure and the zinc-anadinate SBU as well as an illustration of the three-dimensional structure with large cavities can be seen in Figure 3b–e. Other strategies involve the use of low symmetry small biomolecules in order to form cyclic oligomers or post-synthetic covalently attaching biomolecules on the existing MOFs, or encapsulating biomolecules inside the pores by permeation or diffusion [50].

## 3. Polymer/MOF Nanocomposites

Polymer/MOF nanocomposites have attracted wide attention because they combine both the advantages of highly porous MOFs and flexible polymer materials. The combination of MOF with polymers has been reported in a variety of contexts. For mixed-matrix membranes, polymers are often co-blended with MOFs. In composite materials, MOF particles are cross-linked through polymer chains, where some repeating units in the polymer chain act as ligands of the MOF structure. In biomedical applications, MOF nanoparticles are coated with a polymer layer to form core-shell-like architectures. The ideal coating should: (i) be selectively attached on the external surface, avoiding intrusion inside the porous structure; (ii) display suitable stability under physiological conditions; (iii) not interfere with the entrapped drugs; (iv) be obtained in a single step (or few steps), under mild conditions, and (v) enhance the MOF performances for bio-applications by improving their colloidal stability, retarding their degradation, prolonging blood circulation (stealth), and allowing targeting, etc. [60,61].

The polymer coating is generally set up by post-synthetic modification. The strategies developed to coat MOF nanoparticles can be divided in non-covalent and covalent approaches. Non-covalent approaches lie principally on electrostatic interactions or hydrogen bonds. Covalent approaches can be divided in “grafting to” and “grafting from” methods. ‘‘Grafting to’’ involves the reaction of end-functionalized polymers with functional groups located on the MOF, the coordinatively unsaturated metal sites or groups on the ligands, while ‘‘grafting from’’ involves polymerization from active sites on the MOF.

### 3.1. Non-Covalent Attachment

**Figure 4:**
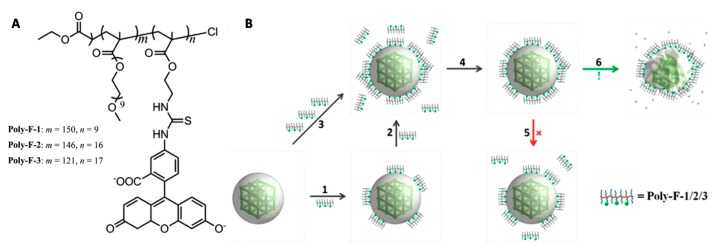
(**A**) Structure of pOEGMA/pAEMA copolymer−fluorescein conjugates (the segment of free AEMA units was omitted for clarification). (**B**) Diagram illustrating the binding/assembly of polymers onto the surface of MIL-101-NH_2_(Fe): (1–3) different concentrations of polymers incubated; (4) free polymers removed by centrifugation; (5) dissociation of polymer from the surface (not observed); (6) degradation of MIL-101-NH_2_(Fe) and redistribution of surface polymers [62].

Liu et al. investigated the non-covalent surface modification of iron(III) carboxylate nano-MOFs with copolymers bearing a fluorescence probe [62]. MIL-101-NH_2_(Fe) bears on its surface positive charges, hydrophobic channels, and open metal sites. It was rationalized that by bearing ionizable carboxylic acid groups, fluorescein (F) would bind to MIL-101-NH_2_(Fe) due to a synergy of electrostatic and hydrophobic interactions. Copolymers comprising of poly(oligoethylene glycol monomethyl ether methacrylate) (pOEGMA) and different amounts of poly(2-aminoethyl methacrylate) (pAEMA) conjugated to fluorescein were prepared (Figure 4A) and a very strong binding affinity to MIL-101-NH_2_(Fe) nanoparticles was observed. Interestingly, it was observed that the binding of the copolymers to MIL-101-NH_2_(Fe) was non-sheddable. In other words, when the free polymers in solution were completely removed, the bound polymers remained bound on the nanoMOFs, instead of partially diffusing into solution (Figure 4B, step 5). It was shown that the surface polymers significantly slowed the degradation of the MIL-101-NH_2_(Fe) nanoparticles, most likely because the diffusion of water in the MOF particles was restricted. Finally, as the degradation of MIL-101-NH_2_(Fe) took place, the amount of polymer adsorbed on the nanoMOFs remained constant, suggesting that it bound to newly formed sites during the degradation of the MOF structure (Figure 4B, step 6).

Azizi Vahed et al. reported the preparation of a novel MOF: MIL-100-metformin(Fe), an antihyperglycemic agent used for the treatment of type II diabetes that also presents anti-cancer properties [63]. In the MOF structure, the metformin molecules are believed to coordinate iron ions, but without bridging two different ions. As they are prone to hydrolysis in aqueous media, MIL-100-Metformin(Fe) nanoparticles were coated to increase their stability. Sodium alginate was chosen to bring about a pH-controlled behavior and formed a complex structure with MIL-100-Metformin(Fe) stabilized through hydrogen bonds. The coated nanoparticles were characterized by Fourier-transform infrared spectroscopy (FT-IR), thermogravimetric analysis (TGA), and x-ray diffraction (XRD) (indicating the MOF crystallinity is retained). The MIL-100-Metformin(Fe) nanoparticles were further loaded with metformin by incubation in a metformin solution, resulting in a total 42% metformin content. The release of metformin was studied at two different pHs and monitored through ultraviolet–visible (UV–Vis) spectroscopy. At pH 1.5 (stomach acidity), the release of metformin was almost negligible (10% in 8 h). In contrast, at pH 8 (intestinal pH), release was much more important (87% within 8 h) and furthermore, no initial burst release was observed. The pH-sensitive behavior was attributed to the carboxylic acid groups of sodium alginate. At low pH, they are protonated and neutral. In basic pH, carboxylate ions are formed, which repel each other due to their negative charge, the polymer expands, and cargo molecules can diffuse out of the MOF nanoparticles.

The same group extended the use of sodium alginate to the coating of ZIF-8, a Zn-based MOF [64]. The coating process was carried out in situ by ball-milling zinc acetate and 2-methylimidazole with sodium alginate. Sodium alginate is believed to coat the particles through interactions between its carboxylate groups and the Lewis acid sites of the framework of ZIF-8 or the functional groups of the linkers. Successful coating was confirmed by infrared spectroscopy (IR), while the similar XRD patterns of the coated and uncoated particles proved that the crystalline structure of ZIF-8 was preserved. Uncoated ZIF-8 particles were loaded with metformin and coated with sodium alginate by immersion. Similar to the release of metformin from the alginate-coated MIL-100(Fe), a negligible release was observed at pH 1.5, while the release was much more important at pH 8.

**Figure 5:**
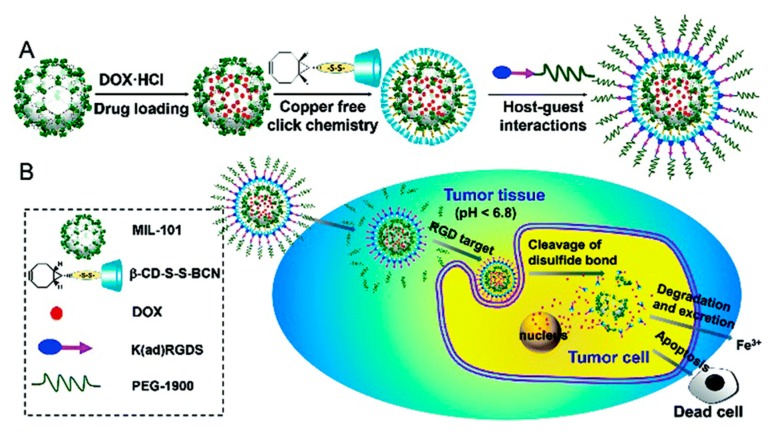
Schematic illustration of (**A**) the drug loading and post-synthetic modification procedure and (**B**) the tumor targeting drug delivery and cancer therapy procedure of the multifunctional MOF based drug delivery system [65].

Combining covalent modifications and non-covalent interactions, Wang et al. reported an interesting smart drug delivery device based on a polymer-coated MOF (TTMOF) bearing stimuli responsive features [65]. Post-synthetic modification of MIL-101-NH_2_(Fe) MOF nanoparticles afforded azide-functionalized MIL-101-N_3_(Fe), which were subsequently loaded with doxorubicin (DOX). Then, the nanoparticles were modified with β-cyclodextrins (β-CD) by a strain-promoted [3 + 2] azide-alkyne cycloaddition reaction between the azide groups of the MOF particles and the triple bond of the β-CD derivatives. Finally, polyethylene glycol (PEG) chains, functionalized with an adamantane group and a lysine-arginine-glycine-asparagine-serine peptide (K(ad)RGDS) for targeting purposes, were attached to the particles through host–guest interactions between the β-CD and the adamantane group of the PEG chains (Figure 5A). The stimuli responsive behavior was implemented through the benzoic-imine bond, which linked the PEG chains to the targeting peptide and a disulfide bond between the β-CD and the MOF nanoparticles.

β-CD were attached to the MOF nanoparticles via a disulfide bond and they blocked the pores, preventing drug release. Indeed, in vitro, less than 15% of the drug was released after a 5-day incubation in phosphate-buffered saline (PBS). However, in the presence of dithiothreitol (DTT), a reducing agent, up to 78% of DOX could be released. This was attributed to the reduction and cleavage of the disulfide bond, resulting in the removal of the β-CD, thus freeing the pore entrances and releasing DOX. In contrast to blood and extracellular fluids, inside the cells, the concentration of glutathione, a biological reducing agent, was 100–1000 times higher, ensuring the rapid cleavage of the S–S bond and the selective, intracellular release of DOX (Figure 5B). Due to the targeting RGD peptide, negligible cellular uptake was observed for non-cancerous cells, but cancerous HeLa cells internalized the nanoparticles; furthermore, uptake was more important at pH 5.0 than at pH 7.4. This is due to the benzoic-imine bond, which linked the PEG chains to the targeting peptide. The benzoic-imine bond was stable at neutral pH. As a result, the targeting peptide was shielded by the PEG chains and the cellular internalization was lower. Under slightly acidic conditions, the benzoic–imine bond was cleaved, the PEG chains were removed, and the targeting peptide was exposed: the outcome was an increased cellular uptake. Finally, the in vivo antitumor efficacy of these nanoparticles was investigated with hepatoma H22 tumor bearing mice (the H22 tumor is integrin positive). Both free doxorubicin and TTMOF nanoparticles exhibited an important tumor growth inhibition, however, side effects, monitored through body weight fluctuations, were considerably lower for TTMOFs.

### 3.2. Covalent Attachment

#### 3.2.1. “Grafting to” Approaches

Zhao et al. reported the successful functionalization of a copper MOF bearing alkynyl functionalized ligands with azide-modified PEG chains via a copper-catalyzed click reaction [66]. Likewise, based on click chemistry but also employing coordination modulation, Lázaro et al. reported on the covalent functionalization of zirconium MOFs through a click modulation strategy. Initially, appropriately functionalized monodentate ligands are introduced in the MOF synthesis, along with bidentate ligands. In the second step, the polymer is installed directly or indirectly on the modulator by a click reaction. Zirconium MOF UiO-66 nanoparticles coated with polyethyleneglycol (PEG) [67], poly(l-lactide) (PLLA), poly(*N*-isopropylacrylamide) (PNIPAM), and heparin [68] were prepared. UiO-66-L1 was synthesized in the presence of modulator L1 bearing an azide moiety, N_3_. Then, employing a copper(I)-catalyzed azide−alkyne cycloaddition (CuAAC), PEG and PLLA polymer chains functionalized with a complementary propargylic moiety, –C≡CH, were covalently bonded to the modulator to produce UiO-66-L1-polymer particles. A slightly different process was adopted for PNIPAM. Starting from UiO-66-L1 via a surface–ligand exchange, modulator L2 bearing a propargylic moiety, –C≡CH, was introduced, followed by click chemistry with an azide-modified PNIPAM polymer.

**Figure 6:**
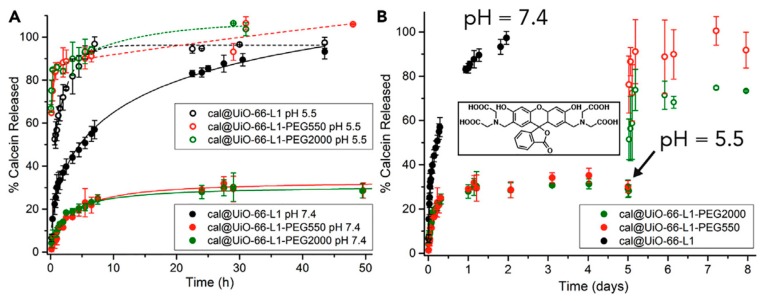
pH-responsive release of calcein from PEGylated UiO-66. (**A**) Calcein-release profiles from UiO-66-L1, UiO-66-L1-PEG550, and UiO-66-L1-PEG2000 in PBS (pH 7.4 and 5.5). (**B**) pH-responsive release of calcein from the PEGylated MOFs. Inset: chemical structure of calcein. Error bars denote standard deviations from triplicate experiments [67].

The attachment of the polymers on the MOF nanoparticles was evidenced through IR, TGA, and mass spectrometry (MS). Powder x-ray diffraction (PXRD) confirmed that the crystallinity of the MOF nanoparticles had not been altered. Scanning electron microscopy (SEM) images showed particles with a more rounded shape and a larger size after the addition of the polymer chains. N_2_ uptake experiments showed that the surface area of the polymer-MOFs had decreased. Dynamic light scattering (DLS) measurements showed that the particles did not aggregate in PBS at pH 7.4. Finally, the polymer–MOFs showed a slower degradation compared to UiO-66-L1/L2. The drug delivery potential of the coated MOF nanoparticles was investigated with calcein as a model drug, and dichloroacetic acid (DCA). The drug was added during the synthesis of the UiO-66-L1/L2 MOFs. It was shown that the drug-loaded nanoparticles were successfully internalized, the endocytosis process depended on the coating, and induced significant cell death. Furthermore, the PEGylated particles showed a pH-responsive behavior, as a faster calcein release was observed at a pH 5.5 compared to 7.4 (Figure 6) [67]. Although some cytotoxicity issues need to be improved, these polymer-coated, DCA-loaded MOFs show promising therapeutic potential.

This strategy was further extended to Zr-fumarate MOFs (Zr-fum): a p-azidomethyl benzoic acid modulator (L1) was introduced in Zr-fum through surface ligand exchange and the azide group of L1 was subsequently used to covalently attach propargyl-terminated PEG chains to the outer surface of the MOFs [69]. Colloidal stability was slightly improved upon PEGylation, and degradation in phosphate buffer saline at pH 7.4 was initially slowed down (induction period) before degrading at a similar rate to non-coated MOF nanoparticles, possibly due to the detachment of the PEG corona. DCA/Zr-fum-L1-PEG exhibited some cytotoxicity toward healthy cells at high concentrations; however, according to the authors, DCA/Zr-fum-L1-PEG had a higher therapeutic efficiency than DCA/UiO-66-L1-PEG.

The modulation strategy was likewise employed by Rijnaarts et al. to introduce PEG chains in MIL-88A MOF particles [70]. Small amounts (0.1–5%) of fumaric acid, an ordinary multivalent ligand in MIL-88A, were replaced by a monovalent PEGylated derivative of succinic acid that acted as a capping ligand, while maintaining a 1:1 stoichiometry between the binding groups. It was shown that the size of the PEG-MIL-88A particles depended on the PEG length and concentration [71]. XRD experiments confirmed that the crystalline structure of MIL-88A was preserved after the insertion of the PEGylated ligand. Elemental analysis evidenced that the PEGylated ligands did not considerably penetrate the bulk of the crystals. The Brunauer–Emett–Teller (BET) surface area decreased probably because the PEG chains blocked the access to the MOF porosity. PEGylated MIL-88A was loaded with sulforhodamine B by counterion exchange. It was observed that encapsulation in the PEG-functionalized particles was more important than in the uncoated particles. This phenomenon was attributed to the higher surface area of the coated particles due to their smaller size/volume.

**Figure 7:**
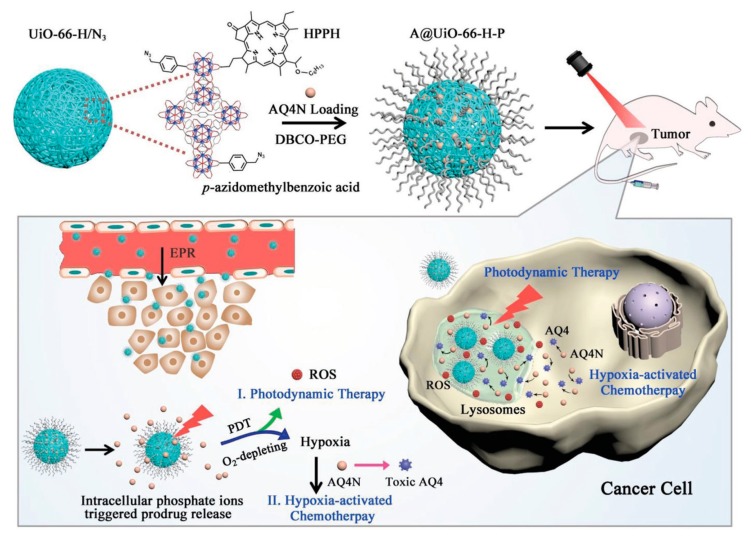
Synthetic procedure of A/UiO-66-H-P nanoparticles and mechanism of photodynamic therapy and hypoxia-activated cascade chemotherapy [72].

He et al. reported nanodevices that would simultaneously co-deliver a photosensitizer necessary for photodynamic therapy and a hypoxia-activated prodrug to implement a combined photodynamic and hypoxia-activated therapy (Figure 7) [72]. The outer surface of the zirconium terephthalate UiO-66 nanoparticles was functionalized with photochlor (HPPH), the photosensitizer, and azide groups, N_3_, by using monocarboxyl photochlor and p-azidomethylbenzoic acid as modulators during the synthesis of UiO-66 nanoparticles. Then, the nanoparticles were loaded with banoxantrone (AQ4N), the hypoxia-activated prodrug. Finally, to improve the stability of the nanodevices, PEG chains were introduced through a simple copper-free click reaction between the azide groups of the p-azidomethylbenzoic acid and alkyne-terminated PEG, DBCO-PEG.

The resultant nanoparticles (A/UiO-66-H-P) were duly characterized and their increased stability in saline solution and low concentration PBS solutions was demonstrated. The nanoparticles efficiently produced reactive oxygen species (ROS), ^1^O_2_, under laser irradiation. It was further shown that the PEGylation had a beneficial effect on the generation rate of ^1^O_2_. In vitro studies demonstrated that the capacity of A/UiO-66-H-P to produce ROS was preserved after the cell internalization of the nanoparticles. The prodrug release studies evidenced a phosphate-controlled release as the release of AQ4N is slow at low PBS concentrations but fast at higher PBS concentrations. The nanoparticles exhibited good biocompatibility and important cellular uptake. In vitro studies with U87MG cells showed that A/UiO-66-H-P inhibited cell growth while in vivo studies showed that A/UiO-66-H-P combined with laser irradiation outperformed any other control therapy.

Zimpel et al. investigated the covalent modification of MOF nanoparticles by exploiting the unsaturated functional groups of the organic linker [73]. This approach allowed for a selective external functionalization, preserving the porous scaffold of the MOF nanoparticles. More explicitly, MIL-100(Fe) nanoparticles were modified by two amino-terminated polymers by coupling the carboxylic acid groups of trimesic acid with the amino groups of the polymers in a carbodiimide-mediated reaction. The two polymers used were an amino-terminated polyethylene glycol and Stp10-C, an oligo-amino-amide bearing a thiol group, which can be further used for the attachment of a fluorescent probe or additional functionalization. XRD and transmission electron microscopy (TEM) confirmed that the crystalline structure of MIL-100(Fe) was retained, the colloidal stability (in water and 10% fetal bovine serum) was significantly increased, a slight decrease of the BET surface area was observed (attributed to the mass increase rather than the loss of porosity); FT-IR and TGA analysis further confirmed the successful attachment of the polymers; and fluorescence correlation spectroscopy (FCS) and DLS measurement showed that the hydrodynamic radius of the particles was 135 ± 45 nm. ^1^H nuclear magnetic resonance spectroscopy (NMR), complemented by some other observations, evidenced the covalent nature of the bond between trimesic acid and the polymers. All of these elements indicated a successful polymer coating of MIL-100(Fe) particles, although the functionalization degree of the nanoparticles was estimated to be rather low. This was attributed to the limited amount of free carboxylic acid groups on the external surface of the MOF nanoparticles. As MIL-100(Fe) is active in magnetic resonance, the relaxivities of the coated nanoparticles were calculated. Albeit having a lower activity than uncoated MIL-100(Fe), visualization of the polymer-coated nanoparticles by magnetic resonance imaging was possible. Finally, Stp10-C-coated MIL-100(Fe) particles were functionalized on the free thiol group of Stp10-C chains with a fluorescent probe, cyanine 5 (Cy5). MIL-100(Fe)/Stp10-C*Cy5 were successfully internalized by murine neuroblastoma N2A cells (as revealed by fluorescence microscopy) and well tolerated.

Marqués et al. used the GraftFast process to covalently coat iron and aluminum trimesate MOF with PEG derived polymeric chains [74]. The process is based on the iron mediated reduction of aryldiazonium salts that generates aryl radicals. The aryl radicals have two roles. First, they react directly with the MIL-100(Fe) particle surface to form a polyphenylene sublayer. Second, they act as initiators for the radical polymerization of acryl-PEG (PEG chains functionalized with acryl moieties). Oligomer chains were formed and, in turn, they reacted with the polyphenylene sublayer to yield the grafted coating layer. This coating occurred in a single step, very quickly, and in aqueous solutions. The PEG coating increased the colloidal stability of the MIL-100(Fe) nanoparticles and slowed down the degradation of the MOF particles without affecting their low cytotoxicity. The coating was found to be rather stable in different aqueous media and under ultrasound sonication. The porosity of the nanoparticles was not affected, and caffeine and tritium-labelled gemcitabine were successfully loaded in the PEG-coated nanoparticles. Finally, it was demonstrated that the PEG coating prevented recognition and removal by macrophages. The GraftFast process was similarly applied to ZIF-8 nanoparticles, with the sole difference being that ascorbic acid was used instead of iron for the reduction of the aryldiazonium salt, thus avoiding the presence of iron-based impurities in the zinc MOFs. As with the MIL-100(Fe) nanoparticles, the colloidal and water stability of ZIF-8 were both increased after coating [75].

In their work, Cai et al. described the coating of Fe-soc-MOF nanocrystals (constructed from oxygen-centered iron carboxylate trimermolecular building blocks and 3,3′,5,5′-azobenzenetetracarboxylic acid as a linker) by polypyrrole (Ppy), resulting in the formation of a core-shell structure [76]. The Fe-soc-MOF nanocrystals were initially modified with functionalized PEG chains bearing a thiol and a carboxylic acid moiety. The PEG chains were attached to the oleic acid, stabilizing the Fe-soc-MOF nanocrystals through their thiol-terminated end via a UV-induced thiol-ene reaction. The resulting nanoparticles were dissolved in a polyvinyl alcohol aqueous solution in the presence of pyrrole monomers. Once pyrrole adsorbed on the surface of the particles, an oxidant was added to initiate an oxidation polymerization to finally obtain Fe-soc-MOF core-shell nanoparticles with a thick Ppy layer (Fe-soc-MOF/PPy). The crystallinity of the Fe-soc-MOF core remained intact after the modifications. However, the Fe-soc-MOF/PPy nanoparticles were almost nonporous because Ppy occupied the porosity of Fe-soc-MOF. Fe-soc-MOF/PPy nanoparticles were characterized by UV–Vis near infrared (NIR) absorption, FT-IR, and TGA, and were found to be stable in PBS solution (37 °C), even after repeated irradiation at 808 nm. It was shown that Fe-soc-MOF/PPy could be used as a T2 contrast agent for T2-weighted magnetic resonance imaging. The nanoparticles (in aqueous dispersions) exhibited photothermal properties, and after a 10-min irradiation at 808 nm, temperatures ranging from 40.6 to 72.4 °C were recorded, depending on the concentration. The photothermal conversion efficiency of thee Fe-soc-MOF/PPy nanoparticles was significantly lower when compared to the pure PPy particles, perhaps due to the small amount of PPy they contained. In vitro, Fe-soc-MOF/PPy nanoparticles had a low toxicity and efficiently inhibited the growth of breast cancer cells (murine breast cancer 4T1 cell line). In vivo studies demonstrated that Fe-soc-MOF/PPy could efficiently convert laser irradiation in thermal energy, and as a result, suppress tumor growth.

Li et al. reported the preparation of a hybrid polymer-MOF architecture for enzyme immobilization [77]. UiO-66-NH_2_ was chosen as the backbone of the structure and post-synthetically, the amino groups were modified into propargylic moieties, –C≡CH. Azide-terminated poly(tert-butyl methacrylate), prepared via atom transfer radical polymerization, was clicked on the alkyne functions of the UiO-66-NH-CH_2_-C≡CH nanoparticles through a copper-catalyzed alkyne-azide cycloaddition. Finally, the tert-butyl protecting groups were removed to yield poly(methacrylic acid) (PMMA)-modified UiO-66-NH_2_ nanoparticles, UiO-66-NH_2_/PMMA. Pectinase was immobilized on UiO-66-NH_2_/PMMA (UiO-66-NH_2_/PMMA/pect) through electrostatic attractions between the carboxylic acid groups of PMMA and the amino groups of pectinase. FT-IR and ^1^H NMR were used to confirm the success of the various modifications. PXRD analysis of all the MOF-containing nanoparticles demonstrated that the crystalline structure of UiO-66-NH_2_ was maintained throughout all the post-synthetic modifications carried out. Additionally, no modifications were observed in the PXRD pattern of UiO-66-NH_2_/PMMA/pect after exposition to a citrate buffer, indicating an increased structural stability in aqueous environments. The colloidal stability after PMMA coating was also increased. The BET surface area decreased considerably, especially the microporosity. This was attributed to the PMMA chains and pectinase molecules covering the pores of UiO-66-NH_2_. Compared to free pectinase, pectinase immobilized on UiO-66-NH_2_/PMMA exhibited an increased stability in acidic and basic media, a good catalytic activity in a wider range of temperatures, and a clearly enhanced long-term stability. Furthermore, UiO-66-NH_2_/PMMA/pect maintained 80% of its catalytic activity after eight continuous recycling cycles.

Nagata and his coworkers capitalized on the thermoresponsive behavior of poly(*N*-isopropylacrylamide) (PNIPAM) to develop a polymer–MOF device for controlled release [78]. PNIPAM-NHS chains were covalently grafted on amino-functionalized UiO-66 crystals to afford UiO-66/PNIPAM. The size of the UiO-66/PNIPAM crystals was around 200 nm, the crystals had an octahedral shape, and their crystalline structure was similar to UiO-66. Based on ^1^H NMR, the modification percentage of the organic ligands grafted by PNIPAM was calculated to be 11%. PNIPAM diffused only slowly in the pores of UiO-66-NH_2_ because of its size; thus, grafting occurred predominantly on the outer surface. The cloud point of PNIPAM was 32 °C. Below 32 °C, PNIPAM is dissolved in water; above 32 °C, it aggregates. Therefore, the pores of UiO-66/PNIPAM were expected to be accessible below 32 °C, but blocked above 32 °C. The temperature-dependent release of guest molecules was investigated using resorufin, caffeine, and procainamide. At 25 °C (coil conformation), the cargo molecules were released within four days. At 40 °C (globule conformation), less than 20% of the cargo molecules were released, even after seven days. After the complete release of guest molecules, UiO-66/PNIPAM could be reloaded, still exhibiting a very similar release behavior to the initial one. Finally, controlled, on-off, stepwise release was demonstrated by switching the temperature between 25 °C and 40 °C every 20 min.

Chen et al. recently reported a polyacrylamide hydrogel coating of UiO-68 zirconium MOF nanoparticles [79]. The polyacrylamide hydrogel was cross-linked through DNA sequences recognizing adenosine triphosphate (ATP). In the presence of ATP, overexpressed in cancer cells, the cross-links dissociated via the formation of ATP complexes and the hydrogel became more permeable, allowing the release of the MOF load. An oligonucleotide was bonded to the linkers of the nanoMOF via a triazine linker, the nucleic acid sequence was hybridized with a complementary one that could interact with the polymer chains of the hydrogel in order to bind the hydrogel to the MOF. The hydrogel coating did not affect the crystallinity of the MOF nanoparticles. The UiO-68 nanoparticles were loaded with Rhodamine 6G fluorophore and doxorubicin before installing the hydrogel layer and ATP-triggered release of the loading was demonstrated.

#### 3.2.2. “Grafting from” Approaches

The typical strategy for covalent modification via the “grafting from” approach is the introduction of bromoisobutyrate moieties on the MOFs and the subsequent polymerization via atom transfer radical polymerization (ATRP), with the MOF particles acting as initiators. For example, Xie et al. employed this strategy to modify the external surface of UiO-66-NH_2_ [80]. The amine groups of the UiO-66-NH_2_ nanoparticles were coupled to α-bromoisobutyryl bromide and the modified MOF nanoparticles were used as multifunctional initiators for the polymerization of poly(ethylene glycol) methyl ether methacrylate (PEGMA). X-ray photoelectron spectroscopy (XPS), XRD, DLS, and SEM confirmed the successful synthesis of the polymer-coated MOF nanoparticles and the retention of the MOF crystallinity and porosity. Interestingly, the modified nanoparticles presented a reversible pH-switchable dispersity in water: clear solutions were obtained at pH 9 and cloudy suspensions were observed at pH 4–7, depending on the length of the PEG chains. This behavior was attributed to the interactions of the PEG chains with the –COOH groups of the superficial partially uncoordinated MOF ligands.

Similarly, Liu et al. grafted copolymers of 2-(2-methoxyethoxy)ethyl methacrylate (MEO_2_MA) and oligo(ethylene glycol) methacrylate (OEGMA) on MIL-101(Al)-NH_2_ [81] where the crystalline structure of MIL-101(Al)-NH_2_ was preserved. Due to the polymer grafting, the polymer-coated MOF particles exhibited a reversible and temperature-dependent hydrophilic/hydrophobic transition. The lower critical solution temperature (LCST) of poly(MEO_2_MA-*co*-OEGMA) was reported to be 39 °C (molar composition: 90% MEO_2_MA and 10% OEGMA). Above the LCST, the copolymer is hydrophobic and insoluble in water; below the LCST, it is hydrophilic and soluble. As a result, stable dispersions of the polymer-coated MIL-101(Al) were observed below 35 °C, and complete precipitation was observed above 45 °C.

Dong et al. reported the synthesis of a dendritic catiomer based on the functionalization of UiO-66-NH_2_ with poly(glycidyl methacrylate) chains [82]. After the polymerization of glycidyl methacrylate, the ring-opening of the epoxide rings with ethanolamine afforded UiO-PGMA-EA bearing a secondary amine and a hydroxyl group. FT-IR and XPS demonstrated the successful synthesis of UiO-PGMA-EA; XRD confirmed the preservation of the structure of UiO-66-NH_2_; and TGA was used to evaluate the amount of grafted polymer. Unlike UiO-66, UiO-PGMA-EA did not aggregate, and due to the hydroxyl groups, exhibited reduced protein adsorption. UiO-PGMA-EA was found to form stable complexes with pDNa due to the abundant amino groups on the PGMA-EA chains, and had high transfection efficiencies, therefore exhibiting potential as a gene carrier for gene therapy [83]. UiO-PGMA-EA was successfully used for the complexation and delivery of mRNA as well as having better performances than the linear PGMA-EA or available commercial products [82].

Likewise, Chen et al. developed an elaborate drug delivery platform founded on the functionalization of zirconium MOF nanoparticles with poly(glycidyl methacrylate) (PGMA) [84]. UiO-PGMA was synthesized as described previously [77], and further reaction of the glycidyl groups with ethylenediamine afforded UiO-PGEDA with two additional amine groups, positively charged at physiological pH. The polymer-coated MOF was loaded with aggregates of doxorubicin (DOX) with a tetraphenylene derivative bearing four –COOH groups (TPE). The DOX-TPE aggregates were used to monitor the DOX release, taking advantage of the fluorescence resonance energy transfer between TPE and DOX. The aggregates were not loaded in the MOF cavities, but were complexed in the polymer layer due to electrostatic attractions. Finally, cucurbit[7]uril (CB[7]) was bound to the residual positively charged amino groups in the polymer layer. CB[7] was used in order to prevent the membrane cell destabilization by the positively charged amino groups of the PGEDA chains, and to regulate the release of DOX. At pH 7, CB[7] and the positively charged amino groups were tightly bound, preventing any DOX leakage. It was demonstrated that at pH 5.0 (endosomal pH), the CB[7] disassembled, allowing DOX release. The empty drug delivery devices showed low cytotoxicity while the DOX-TPE loaded ones had a higher cytotoxicity than free DOX.

Grafting polymers on MOFs is often linked to a decrease in porosity, either because the entrance of the pores is hampered by the polymer chains or because the polymer chains extend into the MOF structure, filling the pores. In response to this drawback, McDonald et al. reported a core-shell MOF architecture with polymer chains grafted on the outer shell, which preserved the core porosity [85]. A shell of IRMOF-3 (zinc ions and 2-aminobenzenedicarboxylate ligand) was grown on a core of MOF-5 (zinc ions and benzenedicarboxylate ligand). Post-synthetic modification of the amino groups of IRMOF-3 with 2-bromoisobutyric anhydride afforded the formation of the initiator for the subsequent polymerization step. Finally, copper mediated atom transfer radical polymerization (ATRP) was carried out using methyl methacrylate as a monomer to yield poly(methyl methacrylate)/IRMOF-3/MOF-5 (PMMA/IRMOF-3/MOF-5). PXRD demonstrated that the crystalline structure of MOF-5 was well preserved throughout all of the modifications. Furthermore, the surface area of PMMA/IRMOF-3/MOF-5 was measured to be 2857 m^2^ g^−1^ and 2289 m^2^ g^−1^, depending on the polymerization duration (5 min and 1 h), showing that the porosity of MOF-5 was intact and accessible. Finally, Raman mapping of the PMMA/IRMOF-3/MOF-5 cross sections showed that the polymer chains are localized on the exterior and within the IRMOF-3 shell.

An interesting functionalization method, lent from the field of mixed matrix membranes, was described by Molavi et al., who modified UiO-66-NH_2_ nanoparticles in order to introduce vinyl moieties and, subsequently grew PMMA chains directly from the surface of the MOF particles [86]. The grafting was confirmed by FT-IR and NMR; PXRD showed that the crystalline structure of UiO-66-NH_2_ was retained; thermal stability was assessed with TGA; and the BET surface area was significantly lower when compared to the vinyl-modified UiO-66-NH_2_ due to the thick and dense PMMA layer that blocked the entrances of the pores. Upon the formation of the polymeric shell, the stability in the PMMA solutions dramatically increased.

Hou et al. used UV-polymerization to graft polymer brushes on MOF particles [87]. The advantage of UV-photoinduced polymerization is that it can be selectively applied to the external surface of MOF particles, thus preserving the porosity of the MOF structure. A suspension of IRMOF-3 particles and methyl methacrylate, styrene, or 2-isopropenyl-2-oxazoline was subjected to UV light. According to the authors, surface radicals are formed on the MOFs by the abstraction of hydrogen atoms and, in turn, these surface radicals initiate a free-radical polymerization. FT-IR was used to evidence the formation of the polymer chains on the MOF particles. PXRD demonstrated that the crystalline structure of IRMOF-3 was not affected by the polymer grafting and TEM images showed the formation of a polymer shell around the nanoparticles. Unlike uncoated IRMOF-3, PMMA-IRMOF retained its crystalline structure even after exposure to air for three days. Besides improving the stability in air, the grafted polymer brushes also prevented the aggregation of the nanoparticles in solution. This method was successfully extended to MOF-5, UiO-66, UiO-66-NH_2_, ZIF-8, MIL-125(Ti), and [Cu(BTCA)_0.5_(H_2_O)_3_]·2H_2_O (BTCA = 1,2,3,4-butanetetracarboxylic acid) particles.

### 3.3. Polymer Coordination to Metal Ions

The superficial metal ions of MOFs in MOF nanoparticles are coordinatively unsaturated, therefore, they can be exploited for the coordination of polymers bearing functional groups with a high affinity to metal ions such as amines or phosphate groups.

Gadolinium nanoparticles have attracted a great deal of attention due to their potential as magnetic resonance imaging (MRI) contrast agents, biosensors, and in drug delivery applications. Rowe et al. reported the covalent modification of gadolinium nanoparticles with interesting, from a biomedical point of view, polymers [88]. The polymers were synthesized via reversible addition-fragmentation chain transfer (RAFT) polymerization, which allows for good control over molecular weights and thus, a low polydispersity index. The RAFT agent used in this work was S-1-dodecyl S′-(α,α-dimethylacetic acid) trithiocarbonate, DATC, and it afforded polymer chains terminated with a trithiocarbonate group. Aminolysis of this group generated a free thiol, which was used to covalently graft the polymers to the Gd MOF (gadolinium 1,4-benzenedicarboxylate) nanoparticles by complexation of the thiolates to the Gd^3+^ ions at the surface of the Gd MOF nanoparticles. The studied polymers were poly[*N*-(2-hydroxypropyl)methacrylamide] (PHPMA), polystyrene (PS), PNIPAM, poly(2-(dimethylamino) ethyl acrylate) (PDMAEA), poly(((poly)ethylene glycol methyl ether) acrylate) (PPEGMEA), and poly(acrylic acid) (PAA) homopolymers. Successful modification of the nanoparticles was confirmed by FT-IR. TEM images showed that the polymers formed a uniform coating on the surface of the Gd MOF nanoparticles. The thickness of the coating depended on the molecular weight of the coating polymer—increased molecular weight afforded increased coating thickness—and could be tuned by varying the polymerization parameters. The polymer coating had good stability and remained intact after several months in aqueous and organic media at room or physiological temperatures. Calculations of the polymer grafting density determined that the coated polymers were in the “brush” regime and a decrease in the grafting density with increased molecular weight of the grafted polymer was observed. Nonetheless, the grafted density values were rather high. The relaxation properties of the unmodified and polymer modified Gd nanoparticles were determined by in vitro MRI and compared with two clinically employed MRI contrast agents: gadopentetate dimeglumine (Magnevist) and gadobenate dimeglumine (Multihance). The Gd MOF nanoparticles modified with hydrophilic polymers exhibited much higher relaxivities compared to the unmodified Gd MOF nanoparticles and Magnevist and Multihance, which is advantageous for their use as clinical positive contrast agents. The relaxivity values tended to increase with increasing polymer molecular weight. In contrast, PS-modified Gd MOF nanoparticles had low longitudinal relaxivity values, attributed to the low water retention due to the hydrophobic nature of PS, and were unsuitable for use as positive contrast agents.

In parallel, aiming at the preparation of novel theragnostic nanodevices, this work was extended to a more elaborate, multifunctional, biocompatible copolymer [89]. Gadolinium 1,4-benzenedicarboxylate nanoparticles were coated with copolymers composed of *N*-isopropylacrylamide, *N*-acryloxysuccinimide (NAOS), and fluorescein O-methacrylate (FMA). FMA was employed for fluorescence tagging of the nanoparticles and NAOS was introduced for further modifications to tailor the nanoparticles for specific applications. Indeed, the polymer backbone was functionalized with a targeting ligand (H-glycine-arginine-glycine-aspartate-serine-NH_2_ peptide (GRGDS-NH_2_)) or a therapeutic agent (methotrexate (MTX), an antineoplastic drug). The polymer coating enhanced the stability of the Gd MOF nanoparticles and growth inhibition studies established an increase in the cell viability in the presence of the coated nanoparticles. The relaxivity properties of the Gd MOF polymer-coated nanoparticles were studied and it was determined that they could produce clinically exploitable results at lower Gd^3+^ concentrations than the contrast agents currently in use, therefore combining fluorescence imaging and magnetic resonance imaging. The targeting potential of these nanoparticles was successfully demonstrated by introducing the GRGDS-NH_2_ targeting pentapeptide on the succinimide groups of the copolymer. Finally, when MTX was attached to the succinimide groups of the copolymer, the MTX-containing polymer-modified Gd MOF nanoparticles inhibited the growth of FITZ-HSA tumor cells, in vitro, likewise free MTX.

Horcajada et al. engineered the surfaces of iron(III) carboxylate MOF nanoparticles through the coordination of amine-bearing polymers to the iron(III) ions of the MOF [21]. Modifications were performed during the synthesis of the nanoparticles or post-synthetically. More specifically, adding alpha monomethoxy-omega-amino poly(ethyleneglycol) (CH_3_-O-PEG-NH_2_) during the synthesis of MIL-88A and MIL-89 afforded the corresponding PEGylated nanoparticles and, similarly, chitosan modified MIL-88A particles were prepared using chitosan grafted with lauryl side chains. MIL-88A was also modified post-synthetically with PEG chains, and MIL-100 was post-synthetically modified with PEG chains and dextran-fluorescein-biotin. MIL-88 and MIL-100 PEGylated nanoparticles were loaded with azidothymidine triphosphate by impregnation and submitted to HIV-activity tests. It was observed that the polymer coating prevented the aggregation of pure MOF nanoparticles in aqueous media without affecting the therapeutic results. When evaluated as MRI contrast agents, the PEGylated nanoparticles showed slightly higher transversal relaxivities than the non-PEGylated ones.

Heparin is a sulfated glycosaminoglycan (polysaccharide) best known for its anticoagulant properties. Heparin coating is expected to confer hydrophilic properties to the surface of the MOF nanoparticles to improve their colloidal stability and to increase blood circulation times, as a result of a lower uptake by macrophages [61]. Bellido et al. studied the surface modification of iron(III) trimesate, MIL-100(Fe), with heparin. Surface modification of MIL-100(Fe) nanoparticles with heparin to afford MIL-100(Fe)/hep was carried out through a simple impregnation of the nanoparticles using a heparin water/ethanol solution. Fast grafting kinetics were observed (around 85% of heparin in solution was associated to MIL-100(Fe) within 4 min), indicating a high affinity of heparin for the MIL-100(Fe) outer surface. Experimental evidence pointed out a coordination of the sulfate groups of heparin to the coordinatively unsaturated iron atoms of MIL-100(Fe). Heparin chains extend partially from the surface in a dense “brush”. The crystalline structure of MIL-100(Fe) was not altered by the heparin coating and no significant changes were observed in the BET surface area, indicating that heparin was only grafted on the outer surface of the nanoparticles and did not block access to the pores of MIL-100(Fe). The robustness of the heparin coating was important in water and cell culture medium, however, this stability was challenged in PBS (serum conditions), most likely because the phosphate groups contained in PBS are stronger complexing groups and replace the sulfate groups of heparin in the iron(III) coordination sphere. The colloidal stability of MIL-100(Fe) nanoparticles was improved, but their hydrolytic degradation was not affected by the heparin coating. These results are in contrast with those reported by Lázaro et al., according to whom, the UiO-66 heparin-coated nanoparticles showed undesirable degradation kinetics and a lower colloidal stability compared to UiO-66 particles [68].

Agostoni et al. reported on the coordination of phosphorylated β-cyclodextrins to the iron ions of MIL-100(Fe) nanoparticles via the coordination of the phosphate groups to the coordinatively unsaturated iron ions of the MOF [60]. It was demonstrated that the phosphorylated β-CD remained at the external surface of the MOF nanoparticles, crystallinity and porosity were preserved, and that the β-CD-coating was very stable in both the phosphate buffer solution and cell culture media, in contrast to the PEG-modified MIL-100(Fe) nanoparticles. Additionally, the authors reported the PEG coating of MOF nanoparticles by the coordination of β-CD-P:Ad-PEG supramolecular assemblies to the external surface of MIL-100(Fe), where β-CD-P:Ad-PEG stands for the host–guest complexes formed by adamantyl-modified PEG chains (Ad-PEG) with phosphorylated β-CD (β-CD-P).

In line with this work, Aykaç et al. reported on the coating of MIL-100(Fe) nanoMOFs with polymeric β-CD derivatives [90]. For this purpose, phosphorylated derivatives of epichlorohydrin crosslinked β-CD polymer were used (polyCD). Modification was carried out by impregnation with aqueous solutions containing the phosphorylated derivative to afford MIL-100/polyCD. The polymers were adsorbed onto the nanoparticles in less than one hour, demonstrating the strong affinity of the nanoMOFs for the phosphorylated polyCD. Isothermal titration calorimetry showed the strong interactions between the phosphate groups of the polymeric CD and the iron trimers in MIL-100(Fe). The number of grafted phosphate groups on polyCD did not seem to affect the interactions with the nanoMOFS, suggesting that only some of the grafted group are accessible to the iron trimers. The nanoMOF particles preserved their morphology, crystalline porous structure, and BET surface area after coating. Rhodamine polyCD was used to assess the stability of the polymeric shell. The more phosphorylated polymeric β-cyclodextrin showed a better stability, with less than 30% detachment after a 24 h incubation in PBS. In order to study the drug loading/releasing capacities of MIL-100/polyCD particles, MIL-100(Fe) nanoparticles were impregnated with 3′-azidothymidine triphosphate before being coated. After one day, a 13% slower releaser was observed for the MIL-100/polyCD nanoparticles when compared to the uncoated ones.

Li et al. described ZIF-8 MOF nanoparticles coated with hyaluronic acid for the pH dependent release of curcumin (CCM), an anticancer drug. CCM-loaded ZIF-8 nanoparticles were synthesized in a one-step process and further embedded in hyaluronic acid with pendant imidazole moieties via the complexation of the imidazole units to the zinc ions of ZIF-8 [91]. According to the XRD measurements, the crystalline structure of ZIF-8 was not affected during drug loading or polymer complexation, the dispersity in aqueous solutions was clearly improved, and TEM imaging, ζ-potential measurement, and ^1^H spectroscopy confirmed the successful preparation of CCM/ZIF-8/HA nanoparticles. In vitro curcumin release was studied at pH 5.5 (tumor tissues pH) and 7.4 (pH of healthy tissues). At pH 7.4, the release of curcumin was slow, and the cumulative release of curcumin was 25% after one week. At pH 5.5, 80% of the loaded curcumin was released within four days. At pH 5.5, the pendant imidazole groups of the hyaluronic acid polymer became protonated and thus were no longer coordinated to the zinc ions. As a result, the protective polymer coating was removed from the ZIF-8 nanoparticles and the degradation of ZIF-8 was faster, resulting in a faster curcumin release. Cytotoxicity was lower for ZIF-8/HA compared to non-coated ZIF-8. Hyaluronic acid binds to the CD44 receptor that is overexpressed by many growing tumor cells. Therefore, compared to free curcumin, an improved cellular uptake was expected, and indeed observed, for CCM/ZIF-8/HA. Furthermore, CCM/ZIF-8/HA induced significant cell death when incubated with HeLa cells.

MIL-100(Fe) nanoparticles were coated by chitosan simply by mixing suspensions of the two materials [92]. The coating was restricted to the outer surface of the MOF nanoparticles and the crystallinity and porosity of the MIL-100(Fe) particles was preserved. X-ray absorption near edge structure spectra of chitosan, MIL-100(Fe) nanoparticles, and chitosan-coated MIL-100(Fe) nanoparticles revealed that the hydroxyl groups of chitosan interacted with the superficial iron ions of the MOF nanoparticles. The chemical stability of MIL-100(Fe) nanoparticles in different physiologic media was increased upon chitosan-coating, however, a general faster aggregation was observed, attributed to the bioadhesive properties of chitosan. The cytotoxicity of the coated nanoMOF was low and the chitosan coating seemed to reduce the inflammatory response and increase the cellular uptake of MIL-100(Fe) nanoparticles.

### 3.4. Encapsulation of MOF into Polymers

Filippousi and her coworkers reported a non-covalent microencapsulation method for the fabrication of MOF/polymer drug delivery devices [93]. UiO-66 and UiO-67 nanocrystals were loaded with either cisplatin (hydrophilic) or taxol (hydrophobic). Drug-loaded MOF were then encapsulated in a poly(ε-caprolactone)–tocopheryl polyethylene-glycol-succinate (PCL–TPGS) copolymer using a solid/oil/water emulsion method. PXRD measurements demonstrated that the crystalline structure of the MOFs was preserved, while the drugs were in an amorphous form. Annular dark field scanning transmission electron microscopy (ADF-STEM) measurements confirmed that the drug-loaded MOF particles were located inside the polymeric microparticles. In vitro drug release studies displayed an enhanced drug-release profile and a smaller initial burst effect when compared to the corresponding drug-loaded MOFs. Finally, as assessed by cell viability data, the polymer coated drug-loaded MOFs nanoparticles were not cytotoxic, even at high concentrations (tested cell lines: U-87 MG (human glioblastoma grade IV; astrocytoma) and HSC-3 (human oral squamous carcinoma)).

**Figure 8:**
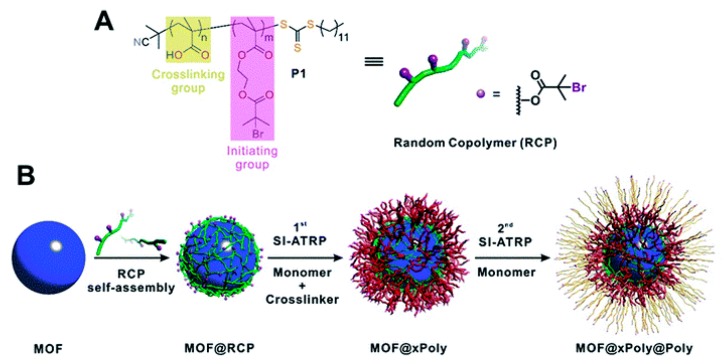
(**A**) Molecular structure of the random copolymer macroinitiator, P1. (**B**) Schematic illustration of typical experimental procedures for growing polymer shells on a MOF particle [94].

He et al. reported an interesting and generalizable strategy for the polymer coating of MOFs (Figure 8) [94]. The MOF nanoparticles were initially embedded in an inter-chain hydrogen bond self-assembled network of a random copolymer. The random copolymer (RCP) contained carboxylic acid groups, as the hydrogen bond directed assembly and the bromoisobutyrate functional groups (Figure 8A). The latter acted as initiators for the atom transfer radical polymerization of a mixture of monomer and cross-linker (e.g., styrene and 1,4-butanediol diacrylate), affording a uniform layer of cross-linked polymer around the MOF nanoparticles. It was demonstrated that polymerization occurred only on the surface of the MOF nanoparticles, the crystallinity and porosity of the nanoparticles were retained, and that the coating was chemically stable, preserving the MOF structure under acidic and basic conditions. The thickness of the polymer coating could be tuned by polymerization time and monomer concentration and it was shown that the wettability of the polymer-coated MOF nanoparticles could be manipulated through the choice of polymer coating. Furthermore, a second polymer layer could be added through a second polymerization step to introduce new functionalities. To demonstrate its wide scope, this surface modification method was applied to UiO-66, ZIF-8, ZIF-67, MIL-96, and MIL-101(Cr) MOF nanoparticles and besides styrene, 2-hydroxyethyl acrylate, n-butyl acrylate, 1H,1H,2H,2H-perfluorodecyl methacrylate, and a mixture of benzyl methacrylate and 1H,1H,2H,2H-perfluorodecyl methacrylate (1/1).

Márquez et al. reported on the fabrication of polymer–MOF patches for cutaneous applications [95]. MIL-100(Fe) caffeine (CAF)-loaded MOF nanoparticles were encapsulated in gelatin from pig skin (GEL) or low molecular weight polyvinyl alcohol (PVA) through a three-step press-molding process: the components of the patches were milled, mixed, and pressed into a wafer. It was found that the patches had a non-bioadhesive character. In release studies, caffeine was progressively released within 48 h from the patches. It is noteworthy that the burst release for the GEL_MIL-100_CAF was only 15%. This was attributed to the lower hydrophilicity and higher strength of GEL compared to PVA, which might slow down the diffusion of caffeine, and the possible coordination of the iron ions of MIL-100 with the amino or carboxylic acid groups of gelatin, which resulted in a more compact network. Overall, polymer-MOF particles exhibited a better release than pure polymer patches, showing the beneficial role of MOF encapsulation. This methodology was successfully extended to ibuprofen.

Liu et al. reported on the modification of hafnium MOF nanoparticles (Hf^4+^ with tetrakis (4-carboxyphenyl) porphyrin) by PEG-grafted poly(maleicanhydride-alt-1-octadecene) [96]. The resulting nanoparticles, thanks to the PEG chains, could undergo dispersion in aqueous media and exhibited rather long blood circulation (blood circulation half-life ca. 3.3 h). The nanoparticles were used for combined radiotherapy and photodynamic therapy and were found to successfully inhibit tumor growth in vivo.

**Figure 9:**
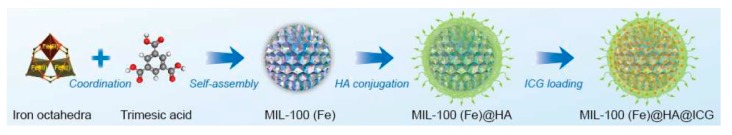
Schematic representation of the synthesis procedure, HA conjugation and ICG loading of MIL-100(Fe) NPs [97].

Cai et al. reported on the use of iron MOF nanoparticles (MIL-100) for photothermal therapy (PTT) [97]. The particles were loaded with indocyanine green (ICG), a photo-responsive organic dye, with an absorption maximum in the NIR, and used for clinical applications. In order to increase the binding affinity selectively for the surface of cancer cells, the nanoparticles were non-covalently conjugated to hyaluronic acid (HA), a natural biopolymer, which was found to mediate the targeting recognition of CD44 over-expressing cancer cells. The MIL-100 coated nanoparticles, MIL-100(Fe)/HA, were obtained simply by mixing the MOF nanoparticles with an aqueous solution of hyaluronic acid, and the ICG molecules were then loaded in the coated nanoparticles to obtain MIL-100(Fe)/HA/ICG nanoparticles (Figure 9). The nanoparticles showed a good colloidal stability in water, and the crystalline structure of MIL-100(Fe) was retained. FT-IR confirmed the successful conjugation with hyaluronic acid. UV–Vis measurements showed that ICG was successfully loaded in the particles and the loading was evaluated around 43 wt %. After encapsulation in the MOF, the ICG absorption and emission maxima were slightly red shifted. When compared to free ICG, MIL-100(Fe)/HA/ICG showed enhanced photostability and photothermal efficiency. Furthermore, it was demonstrated that the MIL-100(Fe)/HA/ICG nanoparticles could be used as MRI contrast agents. According to in vitro studies, MIL-100(Fe)/HA/ICG showed a low cell cytotoxicity, were internalized by CD44-positive MCF-7 cancer cells, and could efficiently kill cells when irradiated at 808 nm (near IR wavelength). When used to treat tumor-bearing mice, PTT was performed 48 h after the injection of the nanoparticles. The temperatures of tumors gradually increased and reached 52 °C after 10 min of irradiation, complete ablation of the tumor was observed after 14 days, and an 80% survival rate was recorded after 20 days.

Different research groups reported polymer coatings by the in-situ polymerization of adsorbed monomers on MOF particles. For example, Wang et al. coated UiO-66 nanoparticles with polyaniline (PAN) for applications in photothermal therapy [98]. Aniline is initially adsorbed on the negatively charged surface of UiO-66 particles because of electrostatic interactions, and then polymerized in situ with the addition of ammonium persulfate as an oxidizing agent. The synthesized UiO-66/PAN nanoparticles were around 100 nm. XRD characterization showed that the crystalline structure of UiO-66 was preserved. The UV–Vis spectrum of UiO-66/PAN nanoparticles dispersed in water showed an absorption around 800 nm, typical of PAN in the form of its emeraldine salt. FT-IR and elemental analysis confirmed the formation of polyaniline on the surface of the UiO-66 nanoparticles. The photothermal performance of UiO-66/PAN were evaluated in vitro and in vivo. In vivo studies were carried out on mice bearing subcutaneous colon cancer xenografts. Upon laser irradiation, the local tumor temperature was between 42 and 45 °C, and the tumor showed complete regression after 10 days.

Wu et al. used polydopamine to aggregate isolated ZIF-8 nanocrystals loaded with glucose oxidase into micrometer-sized particles to facilitate the repeated use of the enzyme [99]. Dopamine was incubated with the enzyme-loaded ZIF-8 at pH 8.5, and polymerized during the incubation. The crystallinity of the MOF was unchanged by the loading and the coating, and though lower activities were observed, higher stability and excellent reusability were demonstrated. Using the same principle, Feng et al. reported on the preparation of MOF nanoparticles for chemo-photothermal therapy [100]. MOF nanoparticles (ZIF-8, UiO-66 and MIL-101) were loaded with doxorubicin (DOX) before being coated with polydopamine, as mentioned earlier. The polydopamine coating was further functionalized with targeting molecules: sgc-8 aptamer and/or folic acid. pH-dependent DOX release was observed, as the ZIF-8 structure is stable at neutral pH but degrades at acidic pH. The coated particles exhibited photothermal activity, attributed to the polydopamine coating. Finally, it was demonstrated that in vivo chemo-photothermal treatment by sgc-8 aptamer-PDA-DOX/ZIF-8 and near infra-red illumination resulted in tumor elimination, without noticeable systemic toxicity and favorable biocompatibility. Yang et al. applied a similar method for the surface functionalization of MOF, aiming to increase the hydrophobicity and water stability [101]. This time, it was based on the easy polymerization of free-base dopamine at room temperature under a mild oxygen atmosphere, affording a polydopamine layer on the MOF. The polydopamine coating was further fluorinated with 1H,1H,2H,2H-perfluorodecanethiol, affording very hydrophobic surfaces. When modified according to this two-step process, HKUST-1 retained its crystalline structure and its stability in water, acidic, or basic media was dramatically increased. Although some amount of dopamine could have potentially penetrated the MOF pores and bound to the Cu ions, in situ diffuse reflectance infrared Fourier transform spectroscopy (in situ DRIFTs) demonstrated that many open metal sites remained after the modification. The polymer loading was optimized in order to retain a high surface area. This modification was successfully extended to ZIF-67, ZIF-8, UiO-66, Mg-MOF-74, MIL-100 (Fe), and Cu-TDPAT.

Likewise, Castells-Gil et al. exploited the easy copper-catalyzed polymerization of catechol derivatives to coat the copper MOF HKUST to improve its moisture tolerance [102]. According to the authors, a ligand exchange took place initially, with the catechols partially replacing the ligands of HKUST on the outer surface of the particles. Then, copper-catalyzed polymerization of catechols was initiated, resulting in the formation of a superficial, tightly bound, thick layer of polymer. Modification with fluorinated 4-undecylcatechol afforded a robust coverage that retained 92% of the MOF porosity and allowed incubation in water for at least one week without significant decrease in crystallinity. The properties of the MOF could be tuned by using appropriately functionalized catechols.

Another strategy to preserve the integrity of the MOF structure and porosity is the coating of MOF particles with microporous organic polymers (MOP). For example, Chun et al. coated UiO-66(Zr)-NH_2_ particles by a microporous organic network assembled from tetra(4-ethynylphenyl)methane and 1,4-diiodobenzene or 4,4′-diiodobiphenyl [103]. The thickness of the coating was controlled by the number of organic building blocks. PXRD demonstrated that the crystalline structure of the UiO-66(Zr)-NH_2_ particles was retained during coating, and coated particles retained their structure when exposed to water. Decrease in the measured surface area was attributed to a partial inclusion of the organic microporous network in the pores of the MOF.

MOF/MOP hybrid nanoparticles (UNP) were prepared by growing a boron-dipyrromethene (BODIPYs)–imine based polymer (by the reaction of 1,3,5-tris(4-aminophenyl)benzene (TAPB) with dialdehyde-substituted BODIPYs (CHO-BDP)) on the surface of pre-synthesized amine-modified UiO-66 MOF seeds (UiO-66 with 50 mol% amino groups), where the amino groups of modified UiO-66 were used as grafting points of the polymer on the MOF particles [104]. The successful formation of the imine polymer was confirmed by FT-IR and solid state ^13^C NMR spectroscopy, while XRD measurements demonstrated that the crystalline structure of modified UiO-66 was preserved. N_2_ sorption experiments evidenced the coexistence of microporous and mesoporous pores within the hybrid nanoparticles, with a calculated BET surface area lying between that of amine-modified UiO-66 MOF and that of pure BDP-imine MOPs. From the view of targeting biomedical applications, folic acid was grafted on the MOP coating by condensation of the amine groups of folic acid with the residual unreacted aldehyde groups of CHO-BDP on the hybrid nanoparticles. No significant cell cytotoxicity was observed for the MOF/MOP hybrid nanoparticles, with or without folic acid. All particles could pass across the cell membrane in the cytoplasm, though cellular uptake was much higher for the folic acid-modified nanoparticles.

Akin to this work, Wang et al. reported the coating of UiO-66 nanocrystals with a cyanine-containing organic polymer built on the surface of UiO-66 particles via a multicomponent Passerini reaction of carboxyl cyanine, o-nitrobenzaldehyde, and 1,6-diisocyanatohexane [105]. Although XRD patterns suggested that the crystalline structure was not affected by the coating, the decreased BET surface area and the increased pore size implied that some structural defects might have occurred on the surface of the UiO-66 crystals. The particles exhibited a good photothermal response and low toxicity, and in vivo tests demonstrated that upon laser irradiation, UiO-66/CyP successfully killed cancerous cells and inhibited tumor growth.

### 3.5. Other Strategies

Finally, to conclude this survey on the modification of MOF particles for biomedical applications, a couple of other interesting strategies will be mentioned. Ostermann et al., aiming at the construction of hierarchical nanostructures, obtained nanofibers of ZIF-8/PVP by electrospinning a PVP/ZIF-8 dispersion [106]. The diameter of the fibers could be adjusted by the polymer concentration. Homogeneous distribution of the nanoparticles inside the fibers was evidenced by microscopy (SEM and TEM) and adsorption measurements showed that the MOF nanoparticles were accessible. The method was further extended to polystyrene and polyethylene oxide.

To address water stability issues, Gamage et al. reported the preparation of MOF-5 composites with polystyrene (PS) [107]. Polystyrene was grafted onto MOF-5 crystals by heating in neat pure styrene at 65 °C. The crystallinity of MOF-5 in the MOF-5-PS composites was not altered by the polymerization of PS. However, the BET surface areas calculated by N_2_ sorption experiments were lower when compared to MOF-5, suggesting that PS chains partially block the pores of MOF-5. Indeed, according to the Raman mapping image and the white-light image, polystyrene is uniformly distributed throughout the MOF-5 crystal. The hydrolytic stability of the MOF-5-PS composites was significantly improved. Dye-adsorption studies showed a decrease of polarity in the pores when PS was grafted. This modification methodology was further extended to other MOFs (IRMOF-3, MOF-177, and HKUST-1) and functionalized styrene monomers (4-bromostyrene). Zhang et al. developed a strategy for the coating of MOF particles with a thin polydimethysiloxane (PDMS) coating [108] and applied it to three different MOFs: MOF-5 with Zn_4_O(COO)_6_ clusters; HKUST-1 with paddle wheel Cu_2_(COO)_4_ centers; and [Zn(bdc)(ted)_0.5_]·2DMF·0.2H_2_O with Zn_2_(COO)_4_N_2_ clusters. The MOF particles were heated in the presence of PDMS, and thermal degradation of PDMS generated volatile silicone oligomers that adsorbed on the MOF and cross-linked to form a PDMS coating. For all of the studied MOFs, retention of the crystalline structure and the porosity was observed after coating and accessibility of the active sites. Water stability was indeed increased as the hydrophobic PDMS coating prevented water molecules from interacting with the metal ions.

**Figure 10:**
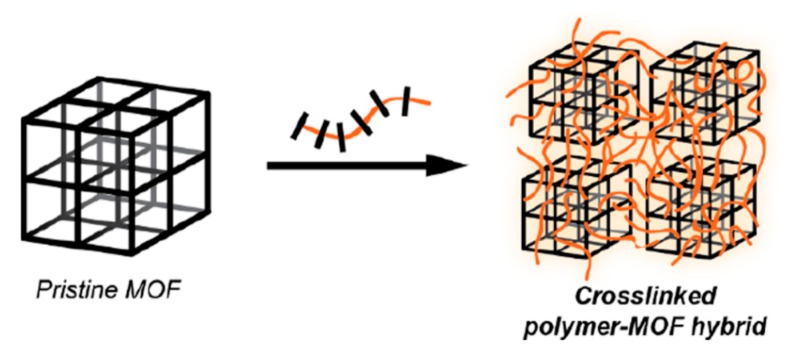
Schematic of direct integration through post-synthetic ligand exchange to form a crosslinked polymer-MOF network with preserved porosity [109].

Pastore et al. reported a different approach that is worth mentioning, although not exploitable for drug delivery application [109]. In this work, poly(amic acid) was conjugated to MOF crystals using post-synthetic ligand exchange (Figure 10). The difference is that the ligand moieties were already incorporated into the poly(amic acid) backbone before the ligand exchange. This strategy was successfully applied to three different polymers, namely MOF-5, ZIF-8 and UiO-66. The crystallinity of the MOF particles was retained and the porosity was preserved, depending on the size of the MOF crystals. It is noteworthy that for micro-crystalline MOF-5, a 99.8% retention of porosity was calculated.

## 4. Concluding Remarks

In summary, we can say that a variety of strategies have been set up for the modification of MOF by polymers to exploit electrostatic interactions and complexation to the metal ions of the MOF structure of covalent grafting. When a MOF is combined with a polymer, its colloidal stability is enhanced without loss of crystallinity. However, a recurrent issue is the decrease of porosity due to the polymer obstructing the entrance to the pores, or the penetration of the polymer chains inside the MOF cavities. Besides increasing the stability, the polymer coating offers the possibility of adding targeting functionalities or introducing a stimuli-responsive release, allowing for the preparation of improved drug delivery or imaging devices.

Up until now, the developed technologies for the use of polymer/MOF and BioMOF nanocomposites are limited due to the lack of knowledge surrounding the interactions of these materials with the human body. In our opinion, the future research efforts should focus, among other things, on the (bio)stability of these systems in physiological conditions and the long-term impact of such compounds on living organisms. Furthermore, since the use of therapeutic proteins is a very promising route in the development of specific drugs, focus should be given to the production of appropriate systems for the delivery of proteins without disrupting their bioavailability and activity. Other critical aspects include biocompatibility issues, batch-to-batch repeatability, pharmacokinetics/pharmacodynamics, dose response, clinical applications, etc.

In conclusion, polymer/MOF nanocomposites constitute a next generation class of multifunctional devices for biomedical applications that can greatly contribute to the improvement of personalized medicine and healthcare in general.
